# 24-Hour survival after cardiopulmonary resuscitation is reduced in patients with diabetes mellitus

**DOI:** 10.15171/jcvtr.2017.30

**Published:** 2017-09-30

**Authors:** Ali Movahedi, Seyed Reza Mirhafez, Hamidreza Behnam-Voshani, Hamidreza Reihani, Gordon A. Ferns, Javad Malekzadeh

**Affiliations:** ^1^Department of Anesthesia and Operating Room Nursing, Neyshabur University of Medical Sciences, Neyshabur, Iran; ^2^Department of Intensive Care Nursing, School of Nursing & Midwifery, Mashhad University of Medical Sciences, Mashhad, Iran; ^3^Department of Basic Medical Sciences, Neyshabur University of Medical Sciences, Neyshabur, Iran; ^4^Department of Pediatrics, School of Nursing & Midwifery, Mashhad University of Medical Sciences, Mashhad, Iran; ^5^Department of Emergency Medicine, School of Medicine, Mashhad University of Medical Sciences, Mashhad, Iran; ^6^Division of Medical Education, Brighton & Sussex Medical School, Falmer, Brighton, Sussex BN1 9PH, UK; ^7^Department of Medical Emergencies, School of Nursing & Midwifery, Mashhad University of Medical Sciences, Mashhad, Iran

**Keywords:** Diabetes Mellitus, Cardiopulmonary Resuscitation, 24-Hour Survival

## Abstract

***Introduction:*** Diabetes mellitus is a risk factor for cardiovascular disease. Some recent studies have shown an association between diabetes and out-of-hospital cardiac arrest incidence and survival. We aimed to investigate whether there is an association between the presence of diabetes mellitus and survival after cardiopulmonary resuscitation (CPR) in patients with an in-hospital cardiac arrest.

***Methods:*** A cross-sectional study was conducted during the period of January to February 2014, among 80 cases of cardiopulmonary arrest in patients at Qaem hospital of Mashhad, Iran. A code 99 was announced after a cardiac arrest was identified, and CPR was performed by the cardiac arrest team. Twenty four hour survival was compared in diabetic and non-diabetic patients who had a return to spontaneous circulation after CPR. We used SPSS statistics for Windows version 16 for data analysis.

***Results:*** The return to spontaneous circulation in the diabetic group was not significantly lower than for the non-diabetic group (42.9% versus 61.0% [*P *= 0.15]). However, the 24-hour survival in the diabetic group was significantly lower than for the non-diabetic group (19.0% versus 44.1% [*P *= 0.04]).

***Conclusion:*** The presence of diabetes mellitus is associated with a significantly lower rate of survival after CPR.

## Introduction

Diabetes is a chronic disease affecting millions of people globally. The prevalence of diabetes mellitus is also increasing, with an estimated 8% of people suffering from diabetes in the United States.^1^ It is expected that 366 million people will have diabetes mellitus globally by 2030.^2^ Cardiovascular disease is the most important cause of mortality in patients with diabetes.^3^ Some studies have shown a significant association between diabetes and the prevalence of primary cardiopulmonary arrest.^4-6^ Cardiopulmonary resuscitation (CPR) is an emergency procedure for patients who have suffered a cardiac arrest.7 Annually more than 400 000 cardiac arrests happened in the United States alone.^8^ The survival rate following CPR is low.^9^ Less than 20% of patients with a cardiac arrest are ultimately discharged from hospital.^10^ The effect of diabetes mellitus on incidence and survival of sudden cardiac arrest in out-of-hospital cardiac arrest patients has been examined in some recent studies.^4-6,11,12^ We aimed to investigate whether there is an association between the presence of diabetes mellitus and survival after CPR in hospital cardiac arrests.

## Materials and Methods

A cross-sectional study was conducted on 80 cardiac arrest patients who attended Qaem general hospital in Mashhad, northeastern Iran during the period of January to February 2014. This educational hospital has 830 active beds. Data for this study were extracted from the database of the study previously reported by Movahedi et al.^13^ All cardiac arrest patients entered into this study if they fulfilled the inclusion and exclusion criteria, detailed below. Inclusion criteria were: in-hospital non-traumatic cardiac arrest, age between 18-85 years, and required intubation. Exclusion criteria were: return of spontaneous circulation (ROSC) before arrival of the CPR team, and severe pulmonary disease. A diagnosis of diabetes mellitus was made either by documentation of this diagnosis in their hospital records, and/or a fasting blood glucose of 126 mg/dL in patients’ blood samples.

When patients in the public ward had a cardiopulmonary arrest, the CPR team was called immediately through automated systems with code 99, and CPR was begun by the existing personal until the arrival of the team. As soon as the CPR team arrived, advance CPR was performed by them. CPR was performed according to the advanced CPR instructions of the American Heart Association in 2010. Chest compression rate was at least 100 per minute and the depth was at least 5 cm and ventilation rate was 8-10 per minute.

ROSC was defined as the existing of a palpable femoral pulse and systolic blood pressure more than 80 mm Hg for longer than 3 minutes. Patients have been under supervision for 24 hours and their 24-hour survival was assessed. Required Information was obtained from patients’ medical records.

All data analyses were performed with the SPSS Statistics for Windows version 16 (IBM Corp., Armonk, NY). Resuscitation outcomes, ETCO2, ROSC, and 24-hour survival were compared between diabetic and non-diabetic groups. The distribution of variables were expressed as mean ± standard deviation (SD) or median (interquartile range). Kolmogorov-Smirnov test was used for compare of distribution of normality of data.

To examine the association of the following pre-arrest and during arrest variables (such as age, sex, length of hospitalization, length of CPR, hypertension, myocardial infarction, ischemic heart disease, ETCO2, mean arterial pressure) on ROSC and 24-hour survival between two groups, we used chi-square test, Fisher’s exact test, independent *t* test and Mann-Whitney test. For investigating the associations between diabetes and CPR-induced 24-hour survival Confounder Factors control, univariate, or multivariate analyses were used. A *P* = 0.05 was considered significant and confidence level was 95%.

## Results

Eighty patients with cardiac arrest were recruited (42 women and 38 men). Of these, 21 patient (25%) were diabetic. The mean age ± SD of the diabetic patients was 71.7 ± 11.7 years and 65.7 ± 16.4 years (*P* = 0.13) in non-diabetic patients. In the diabetic group, 71.4% of patients were female and in non-diabetic group, 45.8% were female (*P* = 0.04). Estimated time from arrest to starting CPR was not significantly different between two groups and was less than 2 minutes (*P* = 0.56). There was no significant difference in duration of CPR process between two groups (*P* = 0.20). Demographic and clinical information of the patients in the two groups are showed in Table 1.

**Table 1:** Demographic and clinical information of the patients in two groups

	**Control (n = 59)**	**Diabetes (n = 21)**	***P*** ** value**
Age (y)	65.7±16.4	71.7±11.7	0.131
Sex, No.(%)			0.043
Male	32(54.2)	6(28.6)	
Female	27(45.8)	15(71.4)	
Length of hospitalization (day)	1.9±1.0	1.8±1.0	0.553
Estimated time from arrest to starting CPR (min)	1.16±0.53	1.09±0.43	0.567
Length of CPR (min)	19.83±9.37	22.85±8.74	0.200
ETCO_2_ (mm Hg)	19.62±11.88	20.71±14.11	0.733
Hypertension, No.(%)	9(15.3)	13(61.9)	0.001
MI, No.(%)	0(0)	3(14.3)	0.003
IHD, No.(%)	7(11.9)	5(23.8)	0.188
MAP after CPR (mm Hg)	71.89±9.94	77.77±25.71	0.275

Abbreviations: Etco_2_, end tidal co_2_; mi, myocardial infarction; ihd, Ischemic Heart Disease; MAP, mean arterial pressure.

ROSC in the diabetic patients was less than for the non-diabetic patients; but this difference was not significant (42.9% in diabetic patients versus 61.0% in non-diabetic patients [*P* = 0.153], odds ratio = 0.47, 95% CI = 0.17-1.31). Multivariate analysis for ROSC was performed by logistic regression and the model was adjusted by confounder factors including age, sex, hypertension and history of CPR ([*P* = 0.179], odds ratio=0.45, 95% CI = 0.26-1.15) (see Table 2).

**Table 2:** Association of diabetes mellitus and Survival after CPR

**Predictor**	**ROSC-**	**ROSC+**	**OR (CI)**_Crude_	*P* ** value**_ Crude_	OR (CI)*	*P* value*
Diabetes, No. (%)	12 (34.3)	9 (20)	0.47 (0.17-1.31)	0.153	0.45 (0.26-1.15)	0.179
Predictor	24h-	24h+	OR (CI)	P value	OR (CI)*	P value*
Diabetes, No. (%)	17 (34)	4 (13.3)	3.34 (1.01-11.16)	0.041	3.01 (1.01-10.49)	0.043

Univariate and multivariate analyses were performed by logistic regression. * The model was adjusted by confounder factors including age, sex, hypertension and history of CPR. The predictors including diabetes and confounding factors in the multivariate model were analyzed as enter method. Abbreviations: OR: odds ratio, CI: confidence interval, ROSC: return of spontaneous circulation; ‏24h+, survived beyond 24 h; 24h-, not survived beyond 24 h‏.

Survival at 24 hours in the diabetic patients was significantly lower than for the non-diabetic patients (19.0% in diabetic patients versus 44.1% in non-diabetic patients [*P* = 0.04], odds ratio=3.34, 95% CI = 1.01-11.16). Multivariate analysis for 24 hour survival was performed by logistic regression and the model was adjusted by confounder factors including age, sex, hypertension and history of CPR ([*P* = 0.04], odds ratio=2.01, 95% CI = 1.01-10.49) (see Table 2).

We also compared underlying diseases between patients who survived after 24 hour and non-survived patients. There was no significant association between underlying diseases and 24 hour survival (Table 3).

**Table 3:** comparison of the underlying disease between survived and non-survived patients

**Underlying disease**	**Number**	**24h-**	**24h+**	***P*** ** value**
CVA	19	14 (73.7%)	5 (26.3%)	0.25
Hypertension	22	16 (72.7%)	6 (27.3%)	0.24
Cancer	12	7 (58.3%)	5 (41.4%)	0.74
MI	3	3 (100%)	0 (0%)	0.28
COPD	5	3 (60%)	2 (40%)	0.62
IHD	12	6 (50%)	6 (50%)	0.50

Abbreviations‏: CVA, central venous accident; MI, myocardial infarction; COPD, chronic obstructive pulmonary disease; IHD, ischemic heart disease; ‏24h+, survived beyond 24 h; 24h-, not survived beyond 24 h‏.

## Discussion

We have found that in patients who underwent CPR, the presence of diabetes mellitus was associated with a reduced ROSC and 24-hour survival. A recent retrospective cohort study^12^ found that diabetic status, prior to arrest, is associated with decreased survival after CPR (*P* = 0.003). Nehme et al^11^ in their study found that diabetes mellitus affects at least one in five patients who have had an out-of-hospital cardiac arrest and is associated with poorer survival and 12-month functional recovery after out-of-hospital cardiac arrest. Jang et al^14^ in a recent study showed that diabetes had a significant negative association with survival after CPR among patients with cardiac diseases, but the association between diabetes and survival after CPR was not significant in patients without cardiac diseases. These studies were conducted in out-of-hospital cardiac arrest patients and our study was conducted on in hospital cardiac arrest patients. Ro et al^6^ in a recent Study found that diabetes mellitus increased the risk of out-of-hospital cardiac arrest. They worked on effect of diabetes on incidence of cardiac arrest. Some other recent related studies^4,5^ also showed this relationship. One study^15^ showed that hospital discharge rate with good neurological outcomes after CPR in patients undergoing out of hospital CPRs was 17.7% in non-diabetic patients, 16.2% in patients with unrecognized diabetes and 9.7% in diabetic patients (*P* = 0.001). One study^16^ on CPR outcomes of dialysis patients showed that there was no significant different between diabetic and non-diabetic patients in hospital discharge after CPR (8% in diabetic and 9% in non-diabetic patients).

This study has some limitations. Diagnosis of diabetes may have been under-estimated. We did not consider the type of diabetes mellitus, its duration, clinical manifestations, and presence of diabetes complications. Many factors can affect the survival rate of CPR. Post CPR care could not be standardized and may be different in patients with and without diabetes mellitus.

## Conclusion

It appears that diabetes mellitus can significantly reduce the survival rate of CPR.

## Ethical approval

This study was approved by Ethics Committee of the Mashhad University of Medical Sciences. Verbal consent was obtained from family members of patients if they were present, and patient’s anonymity and confidentiality was kept. The Ethical Committee Code is 920717 approved by Research Council of Mashhad University of Medical Sciences in 2013-10-09.

## Competing interests

The authors declare there is no conflict of interest.

## Acknowledgments

This study was extracted from a thesis and was sponsored financially by Mashhad University of Medical Sciences. In this regard, we acknowledge Nursing and Midwifery school and Qaem hospital to provide facilities and support for the present study. The authors confirm no conflict of interest.
